# Current and Historical Resource Nitrogen Supply Affects the Eco‐Physiological Traits and the Ionome of a Diazotrophic Cyanobacterium

**DOI:** 10.1111/ele.70051

**Published:** 2024-12-31

**Authors:** Nicole D. Wagner, Clay Prater, Caleb J. Robbins, Felicia S. Osburn, Jingyu Wang, Punidan D. Jeyasingh, J. Thad Scott

**Affiliations:** ^1^ Department of Biological Sciences Oakland University Rochester Michigan USA; ^2^ Department of Integrative Biology Oklahoma State University Stillwater Oklahoma USA; ^3^ Department of Biological Science University of Arkansas Fayetteville Arkansas USA; ^4^ Center for Reservoir and Aquatic System Research Baylor University Waco Texas USA; ^5^ Department of Biology Baylor University Waco Texas USA; ^6^ Department of Biology University of Central Arkansas Conway Arkansas USA; ^7^ College of Environmental Science and Environmental Engineering Zhejiang University of Water Resources and Electric Power Hangzhou China

**Keywords:** ecological stoichiometry, HABs, N‐fixation, photosynthetic pigments

## Abstract

Diazotrophic cyanobacteria can overcome nitrogen (N)‐limitation by fixing atmospheric N_2_; however, this increases their energetic, iron, molybdenum, and boron costs. It is unknown how current and historic N‐supplies affect cyanobacterial elemental physiology beyond increasing demands for elements involved in N‐fixation. Here, we examined the changes in pigment concentrations, N‐storage, and the ionome (i.e., multivariate elemental composition) of the freshwater diazotroph *Dolichospermum flosaquae* adapted to an N‐gradient for two temporal scales: 27 days and 45 months. We found short‐term adaptation of *Dolichospermum* to low N‐supply decreased pigment concentrations, N‐storage, N:carbon (C), and increased boron:C, calcium:C, and magnesium:C than high N‐supply adapted populations. *Dolichospermum* adapted to low N‐supplies for 45 months had higher pigment concentrations, N‐storage, and lower boron:C, calcium:C, magnesium:C, and phosphorus:C than the short‐term adapted populations when grown in low N‐supplies. Our results highlight the connections between the ionome and physiology, identifying the previously unrecognised roles of elements that can be used to advance physiological patterns.

## Graphical abstract

Diazotrophic cyanobacteria can overcome nitrogen (N)‐limitation by fixing atmospheric N2; however, this comes with increased energetic, iron, molybdenum, and boron costs. Here, we examined the changes in pigment concentrations, N‐storage, and the ionome of the freshwater diazotroph *Dolichospermum flosaquae* adapted to an N‐gradient for two temporal scales: 27 days and 45 months. Our results highlight the connections between the ionome and physiology, identifying the previously unrecognised roles of elements that can be used to advance physiological patterns.

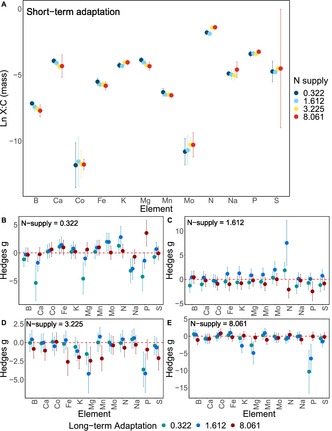

## 1 Introduction

Biological nitrogen (N)‐fixation is a fundamental ecological process performed exclusively by certain prokaryotes (diazotrophs) that supplies new bioavailable N to ecosystems. Diazotrophy evolved approximately 3–3.5 billion years ago and has been maintained in the freshwater cyanobacteria orders Nostocales and Stigonematales (Sánchez‐Baracaldo et al. 2022). Some of these diazotrophs can form harmful cyanobacterial blooms that can persist in N‐deficient conditions, often trading off growth rates for N‐assimilation (Osburn, Wagner, and Scott 2021; Scott, McCarthy, and Paerl 2019). While diazotrophic taxa are hypothesised to dominate the phytoplankton community in low N:phosphorus (P) lakes (Smith 1983), they are increasingly observed in high N:P lakes (Sterner et al. 2020). Diazotrophic cyanobacterial traits and metabolism are highly dependent on the external environment (Osburn, Wagner, and Scott 2021; Wagner et al. 2023; Wang et al. 2021), which causes substantial rearrangement of biochemical pathways (e.g., Harke and Gobler 2015) that influence the cell quotas and fluxes of multiple other elements (e.g., Ipek and Jeyasingh 2021).

In N‐deficient environments, heterocystous diazotrophic cyanobacteria begin to fix atmospheric N_2_ within a day of combined N source depletion (Herrero et al. 2004). A biochemical cascade activates N‐fixation by first transforming vegetative cells into heterocysts (Herrero, Stavans, and Flores 2016), which protect the N‐fixing enzyme, nitrogenase, from oxygen (Fay 1992). Heterocysts maintain a low‐oxygen environment by the absence of the oxygen‐producing photosystem II (PSII), increasing respiration rates, and decreasing oxygen diffusion through a thicker cell membrane (Herrero and Flores 2019). The nitrogenase enzyme can account for up to 10% of the total protein pool (Raven 2012), which consequently increases the demand for iron (Fe) and molybdenum (Mo) that are the catalytic centres of the enzyme (Flores et al. 2005). Thus, depending on the quantity of nitrogenase contained within heterocysts, micronutrient demands can be dramatically altered compared to diazotrophs growing on adequate supplies of dissolved inorganic or organic N sources.

There are ~20 biogenic elements required for life in plants and animals (Fraústo da Silva and Williams 2001). Elements are used in biomass production (carbon [C], N, P, and sulphur [S]); to balance charges (sodium [Na], potassium [K], calcium [Ca], magnesium [Mg]); and in catalytic processes (Fe, Mo, cobalt [Co], manganese [Mn]). Consequently, the elemental composition of cells reflects the evolutionary history and physiological status of an organism (Huang and Salt 2016). Different forms of nutrient stress can alter the collective elemental composition (hereafter, the ionome) in complex, yet predictable ways (e.g., Jeyasingh et al. 2020; Salt, Baxter, and Lahner 2008). Attention to the biochemical roles played by each of the biogenic elements can thus provide additional information that is useful for biological inference. For example, Jeyasingh et al. (2023) found that elements involved in bacterial biochemical catalysis (i.e., Fe and Mn) were the most sensitive to nutrient stress, followed by major ions that maintain electrochemical balance (e.g., Ca, K, Mg, Na), and then the biomass elements (C, N, P) that make up the ionome. Besides the increased Fe and Mo demands involved in N‐fixation, we know little about the ionomic responses of diazotrophic cyanobacteria to changes in N‐supply, particularly in respect to other key functional traits.

The physiology of cyanobacteria is shaped by current and previous environmental conditions. Physiological adjustments to environmental change occurring within a few generations (i.e., short‐term adaptation) have been particularly well studied in the context of light‐harvesting mechanisms (Schagerl and Müller 2006; Wang et al. 2021) and elemental composition of cyanobacteria (Ipek and Jeyasingh 2021; Osburn, Wagner, and Scott 2021; Wagner et al. 2023; Wang et al. 2021). Additionally, there are physiological changes associated with long‐term adaptation to environmental conditions. The harmful cyanobacterial bloom‐forming genus, *Microcystis*, evolved in higher temperatures exhibits faster growth rates and higher cyanotoxin concentrations than *Microcystis* evolved at moderate temperatures (Layden et al. 2022). However, long‐term adaptation to environmental conditions is often associated with physiological trade‐offs. For example, *Microcystis* that can thrive under salt‐stress had significantly slower growth rates in low‐salt environments (Melero‐Jiménez et al. 2019). Furthermore, *Dolichospermum* populations adapted to low N:P conditions for 4 months fixed more N when grown in low N:P environments, which did not come at the cost of growth (Osburn, Wagner, and Scott 2021). While short‐ and long‐term adaptations alter nutrient demands and elemental composition of cyanobacteria, it remains elusive how adaptation affects the interactions between the ionome and physiological traits.

Here, we examine how N‐supply affects the diazotrophic cyanobacterium *Dolichospermum flosaquae* at different temporal scales (i.e., over 27 days, and 45 months). For the purposes of this manuscript, we ascribe responses to the 27‐day experiment to short‐term adaptation, and the 45‐month experiment to long‐term adaptation. We hypothesise that altering the N‐supply will invoke physiological responses that result in unique ionomes in the short‐term adapted *Dolichospermum* populations. We predict that *Dolichospermum* populations grown in low N‐supply will have an N‐fixation phenotype that would cause an N‐fixation growth trade‐off; higher cumulative N‐fixation rates; lower C standardised photosynthetic pigments (Wagner et al. 2023; Wang et al. 2021); and higher Fe:C, B:C, and Mo:C (Ipek and Jeyasingh 2021; Kelly et al. 2021; Whittaker et al. 2011) than *Dolichospermum* populations grown in high N‐supply conditions. Additionally, we hypothesise that long‐term adaptation to low N‐supplies will enhance the expression of the N‐fixation phenotype caused by the continuous demand of N‐fixation. We predict that long‐term adaptation to low N‐supplies will have a phenotype primed for N‐fixation that includes more Fe, Mo, light harvesting pigments, and more N‐storage than short‐term adapted populations grown in low N‐supplies. While we acknowledge that our predictions are solely based on previous literature (Ipek and Jeyasingh 2021; Wagner et al. 2023), we expect to find novel connections between the ionome and physiology that could not be predicted based on only changes in the elemental composition of the physiological pools (i.e., boron [B] in N‐fixing heterocystous cyanobacteria; Bonilla, Garcia‐Gonzalez, and Mateo 1990).

## 2 Materials and Methods

### 2.1 Maintenance of Short‐Term Adaptation Experiment Stock Culture

**FIGURE 1:**
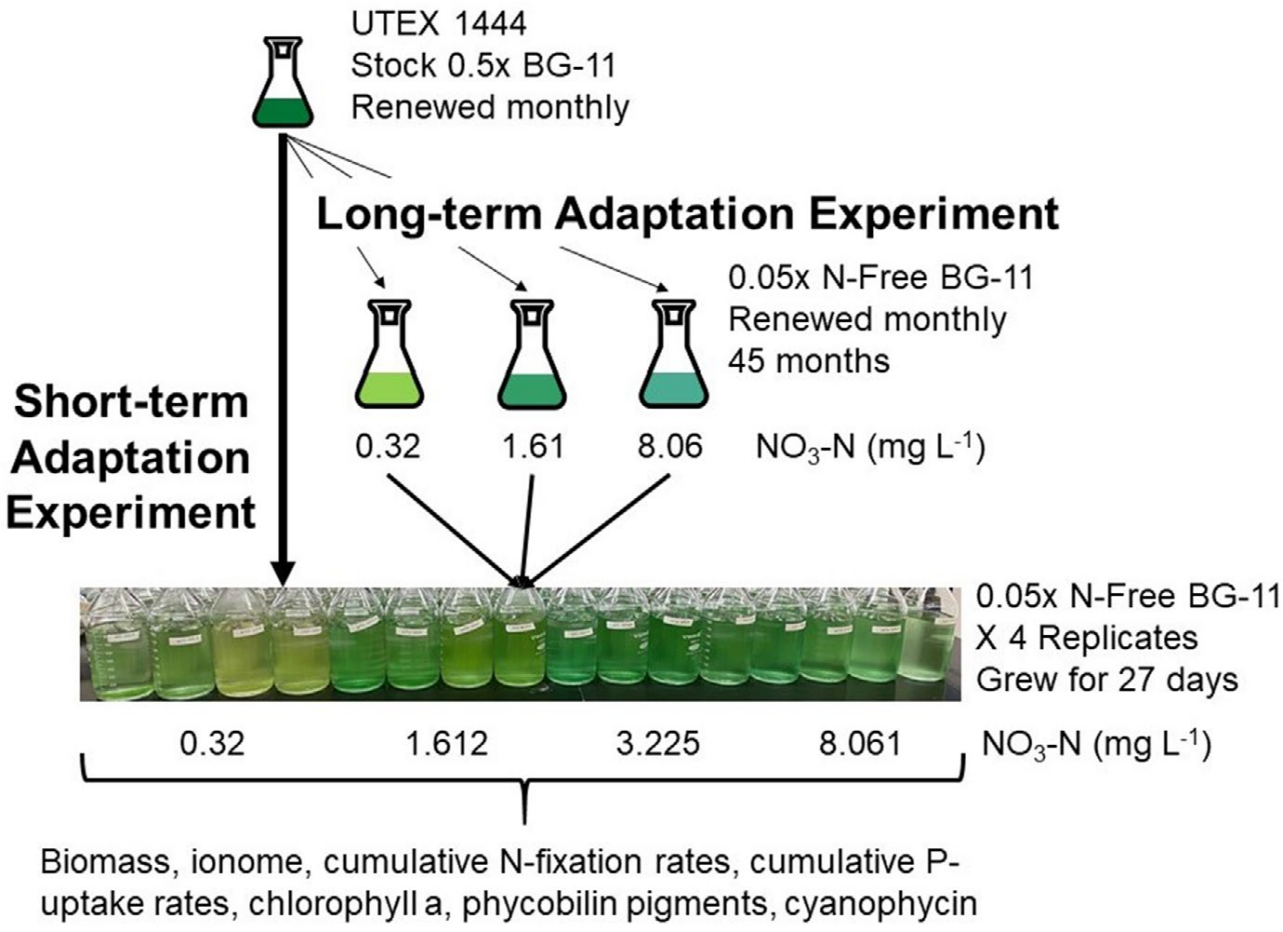
Conceptual experimental design. The 0.5× BG‐11 stock was divided into three long‐term adaptation stocks that varied in the amount of nitrogen (N) supplied. These long‐term adaptation populations were renewed monthly for 45 months prior to the common garden experiment. In coordination with the long‐term adaptation experiment, we also include a short‐term adaptation experiment that originated using the UTEX 1444 stock grown in 0.5× BG‐11. The long‐ and short‐term adaptation experiments were grown for 27 days under different N‐concentrations, F with each N supply replicated four times.

Non‐axenic *D. flosaquae*, UTEX 1444, was purchased from the University of Texas Culture Collection and maintained at Baylor University for over 48 months. Stock *D. flosaquae* cultures were grown in 500 mL borosilicate Erlenmeyer flasks containing sterilised 0.5× BG‐11 (Sigma‐Aldrich; see Table S1 for other element concentrations) at 26°C with a light intensity of 120 μmol m^−2^ s^−1^ on a light:dark cycle of 14 h:10 h. Cultures were renewed every 4 weeks by transferring 1% of the stock culture to new sterile 0.5× BG‐11 media (Figure 1).

### 2.2 Maintenance of Long‐Term Adaptation Experiment Stock Cultures

After 12 months of renewing the short‐term adaptation culture, in June 2017, we split the maintenance stock culture into three long‐term adaptation subcultures that varied in N‐supply. Each long‐term adaptation subpopulation (*n* = 1) was grown in 500 mL borosilicate Erlenmeyer flasks containing 5% or 20× diluted N‐free BG‐11 (see Table S1 for element concentrations) with nitrate‐N added to generate the N‐supply of 0.322, 1.612, and 8.061 mg L^−1^. The subcultures were renewed ~monthly by transferring 1% of the subculture to new sterilised media containing the appropriate N‐supply. Nickel (Ni) is a required trace‐metal for hydrogenases that recycle the H^+^ ions used by the nitrogenase enzyme (Bothe et al. 2010). Although we did not measure Ni because it is not one of the trace elements in BG‐11, we suspect that Ni could have been added as a contaminant of one of the other trace elements or from the use of the borosilicate flasks. The long‐term adaptation of *Dolichospermum* subpopulations to the different N‐supplies lasted for a total of 45 months (Figure 1). It is likely each population experienced a different number of generations over the 45 months caused by differences in growth rates among the N‐supply treatments. Extrapolating from Osburn, Wagner, and Scott (2021), we estimate the long‐term adaptation subpopulations underwent ~ 600 generations.

### 2.3 Experimental Design

The short‐ and long‐term adapted populations were grown in a common garden factorial experiment across an N‐supply gradient of 0.322, 1.612, 3.225, and 8.061 mg L^−1^ with each population having four replicates per treatment (Figure 1). Out of the resulting 64 experimental units, 16 were short‐term adaptation and the remaining 48 were the long‐term adaptation *Dolichospermum* populations. All populations were grown in 850 mL batch cultures which consisted of 20× diluted N‐free BG‐11 (see Table S1 for elemental concentrations), and a ratio of ^15^N/^14^N of nitrate‐N that resulted in a δ^15^N of 100‰ relative to air. We subsampled from each resource N‐supply jar to obtain the average initial nitrate‐N and soluble reactive P (SRP) concentration for each resource N‐supply treatment regardless of short‐ or long‐term adaptation. To each experimental unit, we added 0.05 mg L^−1^ of particulate C biomass from the corresponding short‐ or long‐term adaptation populations. All 64 batch cultures were transferred into an incubator at 26°C with a light intensity of 120 μmol m^−2^ s^−1^ on a light:dark cycle of 14 h:10 h. Cultures were shaken daily to prevent settling and randomly relocated in the incubator to prevent location artefacts. Every other day a 2 mL subsample of each culture was removed to monitor in vivo chlorophyll a (Chl a; Trilogy Fluorometer, Turner Designs, Figure S1). This monitoring indicated the cultures remained in exponential growth throughout the experiment with the exception of 1.612 mg L^−1^ resource N‐supply (Figure S1). We speculate that resource 1.612 mg L^−1^ N‐supply was still growing but did start to form visible clumps that could have affected the in vivo Chl a measurements during the last week of the experiment.

After 27 days of growth for the short‐ and long‐term adapted populations, we measured photosynthetic efficiency and filtered each population for C, N, P, phycobilin pigments, and Chl a on 0.7 μm glass fibre filters (Whatman GF/F). Samples for δ^15^N analysis were collected on 0.7 μm quartz filters (Whatman), and cyanophycin and ionome samples were collected on 0.45 μm cellulose acetate filters. The 0.45 μm filtrate was collected to analyse dissolved inorganic nitrogen (DIN) and SRP. All filters and filtrate were frozen at −20°C until analysed.

### 2.4 Elemental Analysis

We dried filters for CN analysis at 60°C for 24 h prior to analysis on an elemental analyser (FlashSmart NC Soil, ThermoFisher) as previously described (Wagner et al. 2023). Filters for particulate P were digested using a hot solution of 3% w/v potassium persulphate (APHA 1992) and analysed as previously described (Wagner et al. 2023). Samples for ionomic analysis were dried and digested in a 2:1 (v/v) solution of 70% trace clean nitric acid and hydrogen peroxide solution in polypropylene tubes for 3 days. After extraction, the samples were diluted with MilliQ water and analysed on an inductively coupled plasma optical emission spectrometer (ICP‐OES; Thermo Scientific icAP 7400). Samples were run with an in‐line internal standard of yttrium (CPI International, Santa Rosa, CA) to correct for instrument drift and potential of matrix effects. Additionally, the ICP‐OES was calibrated with an external calibration standard reference solution (CPI International, Santa Rosa, CA). We found that B, Ca Co, Fe, K, Mg, Mo, Mn, Na, and S were consistently above the detection limits in all treatments and included in the analysis (Table S2).

### 2.5 Dissolved Nutrients

Initial and 27‐day SRP and DIN samples were analysed on a Lachat 8500 flow‐injection auto‐analyser with an ASX‐520 autosampler (Hach, Loveland, CO; APHA 1992). We averaged the initial SRP by resource N‐supply and used the final SRP from each experimental unit to calculate P‐uptake rates using the following equation:

(1) $$P\;\text{uptake rate}=\frac{\left(P_{\text{initial}}-P_{\text{final}}\right)}{h}$$

Where all nutrient concentrations are in μg L^−1^ and 648 h of the 27‐day experiment, thus giving nutrient uptake rates in μg L^−1^ h^−1^.

### 2.6 Cumulative N Fixation Rates

Quartz filters for cyanobacteria δ^15^N analysis were dried for 24 h prior to analysis on an elemental analyser (Costech 4010) attached to an isotope ratio mass spectrometer (Thermo‐Electron Delta V Advantage), with the δ^15^N standard reference as air (0‰). We used the same approach as Taylor et al. (2023) to calculate cumulative N_2_ fixation rates based on the ^15^N stable isotope described in the Supporting Information  S1.

### 2.7 Photosynthetic Efficiency and Light Harvesting Pigment Analysis

We calculated photosynthetic efficiency (FvFm) as previously described by Parkhill, Maillet, and Cullen (2001) and additional details in the Supporting Information. FvFm calculates the proportion of reaction centres that are open and available for light harvesting compared to reactions centres that are closed with the energy being dissipated as heat. Phycobilin pigments (PBPs) were extracted using 5 mL of 0.1 M phosphate buffer pH 7 and analysed on a UV/Vis spectrophotometer as previously described (Wagner et al. 2023; Wang et al. 2021). Chl a was measured according to the EPA 445.0 method (Arar and Collins 1997) and described previously (Wagner et al. 2023). Cyanophycin, an N storage molecule, was analysed as previously described (Li et al. 2001; Liu and Yang 2014) and additional details are available in the Supporting Information.

### 2.8 Statistical Analysis

All analyses were completed in R version 4.2.3 (R Core Team 2023) and data and R scripts used for analysis are archived in FigShare at DOI: https://doi.org/10.6084/m9.figshare.24312436.v4. We examined the eco‐physiological and ionome effects of short‐term adaptation to different N‐supplies using generalised linear regression (see Supporting Information for details). To compare proportional differences in elemental composition across treatments, all elemental data were transformed into additive log ratios (Aitchison 1982) by calculating the natural log of each element in a sample (*X* = 11) divided by a common elemental denominator (i.e., ln(*X*:P); Aitchison 1982). Rather than selecting this denominator based on historical convention (i.e., P or Co), we used Procrustes analysis to determine which set of log ratios best explained total log ratio variance (Greenacre, Grunsky, and Bacon‐Shone 2021). The ionome was standardised using the additive log ratio (ALR) transformation with all elements standardised to C (Aitchison 1982). We chose to standardised everything to C for two reasons, our pigments are standardised to C, and we identified C as the best denominator after performing a Procrustes analysis that compares the total log ratio variance explained by ALRs calculated using all possible denominators in our dataset (Greenacre, Grunsky, and Bacon‐Shone 2021). Thus, the ionome is presented and analysed as the natural log of the element:C mass ratio.

We examined the eco‐physiological and ionome effects of long‐term adaptation to different N‐supplies by computing an effect size estimate between the long‐ and short‐term adaptation responses within an N‐supply treatment. The effect size was estimated using the hedges_g() function within the effect size package and 95% confidence intervals (Ben‐Shachar, Lüdecke, and Makowski 2020). If the 95% confidence interval does not overlap with zero, there was a significant difference in the long‐term adaptation population compared to the short‐term adaptation population for that resource N‐supply. Negative effect sizes indicate the long‐term adaptation population has a lower response than the short‐term adaptation, and vice‐versa.

Due to the physiological responses being influenced by resource N‐supply, we explored which elements were correlated to the physiological traits by grouping all the data from the short‐ and long‐term adaptation populations. We regressed the physiological responses against natural logged transformed multi elemental ratios containing elements found to be the strongest multivariate predictors of each response. Further details are provided in the Supporting Information. When more than two elements were identified, we created an amalgamated ratio by summing all the elements with lower proportion in the numerator and dividing by the element in greatest quantity in the denominator (Greenacre, Grunsky, and Bacon‐Shone 2021). The FvFm, PBP, Chl a, and cyanophycin were log‐transformed before analysis to meet the linear regression assumptions. Regardless of transformation for the analysis, all data are graphed using non‐transformed scaling.

## 3 Results

**FIGURE 2:**
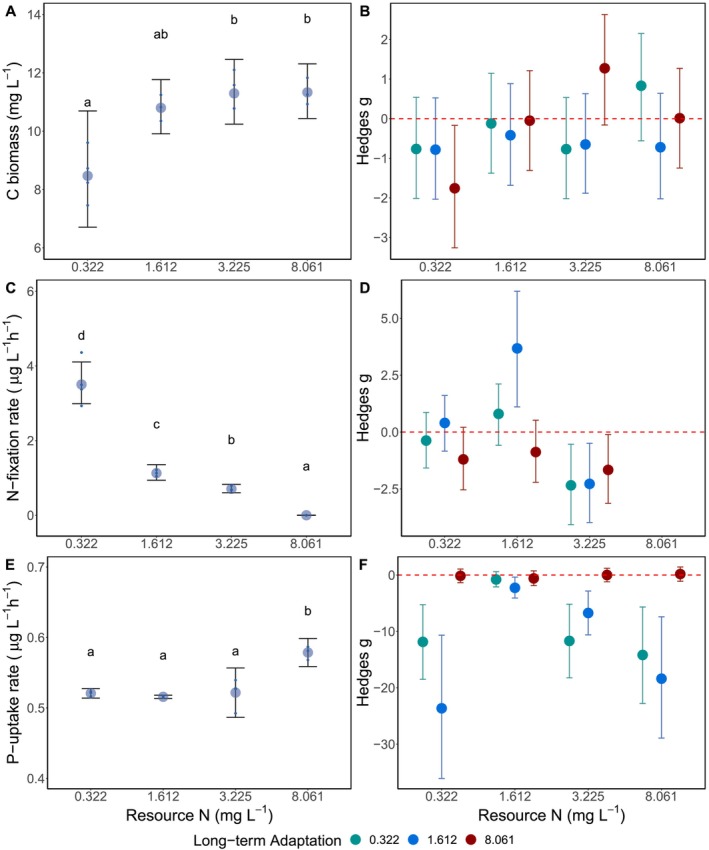
*Dolichospermum* population biomass (A), cumulative nitrogen (N)‐fixation rate (μg L^−1^ h^−1^; C), and cumulative phosphorus (P)‐uptake rate (μg L^−1^ h^−1^; E) grown across an N‐supply gradient (mg L^−1^) for the short‐term adaptation experiment. Letters indicate significant grouping using the Sidak post hoc comparisons. *Dolichospermum* populations adapted to different N supplies (0.322 mg L^−1^; green, 1.612 mg L^−1^; blue, and 8.061 mg L^−1^; red) long‐‐term were compared to the short‐term adaptation using the Hedges *g* effect size and 95% confidence intervals for biomass (B), cumulative nitrogen (N)‐fixation rate (D), and cumulative phosphorus (P)‐uptake rate (F). If 95% confidence interval overlaps with the 0 (dashed red line) it indicates the long‐term adaptation was not different from the short‐term adaptation grown under the corresponding N supply concentrations. If the 95% confidence interval does not overlap with the red dashed line, it indicates the long‐term adaptation response differed from the short‐term adaptation response. Negative effect sizes indicate the long‐term adaptation response was less than the short‐term adaptation response, and positive effect sizes indicate the long‐term adaptation response was greater than the short‐term adaptation response.

C biomass was approximately 25% lower in the lowest N treatment compared to the other N‐supplies for the short‐term adaptation (*F*_3,7_ = 8.73, *p* = 0.009; Figure 2A). Long‐term adaptation to other N‐supplies did not systematically affect the biomass, and only minimal changes were observed (Figure 2B). Cumulative N‐fixation rates for the short‐term experiment were negatively related to N‐supply (*F*_3,7_ = 3924, *p* < 0.001; Figure 2C). Long‐term adaptation to different N‐supplies affected cumulative N‐fixation rates. The population adapted to 1.612 mg L^−1^ N long‐term had a significantly higher N‐fixation rates when grown in the same N‐supply than the short‐term adapted populations (Figure 2D). All long‐term adaptation populations had lower N‐fixation rates under 3.225 mg L^−1^ N‐supply (Figure 2D). Cumulative P‐uptake rates for the short‐term experiment was approximately 9% higher in the 8.051 mg L^−1^ N compared to the other N‐supply populations (*F*_3,7_ = 47.12, *p* < 0.001; Figure 2E). In contrast, populations adapted to the two lowest N‐supplies long term had lower cumulative P‐uptake rates for all N‐supplies except the 1.612 mg L^−1^ N treatment (Figure 2F).

**FIGURE 3:**
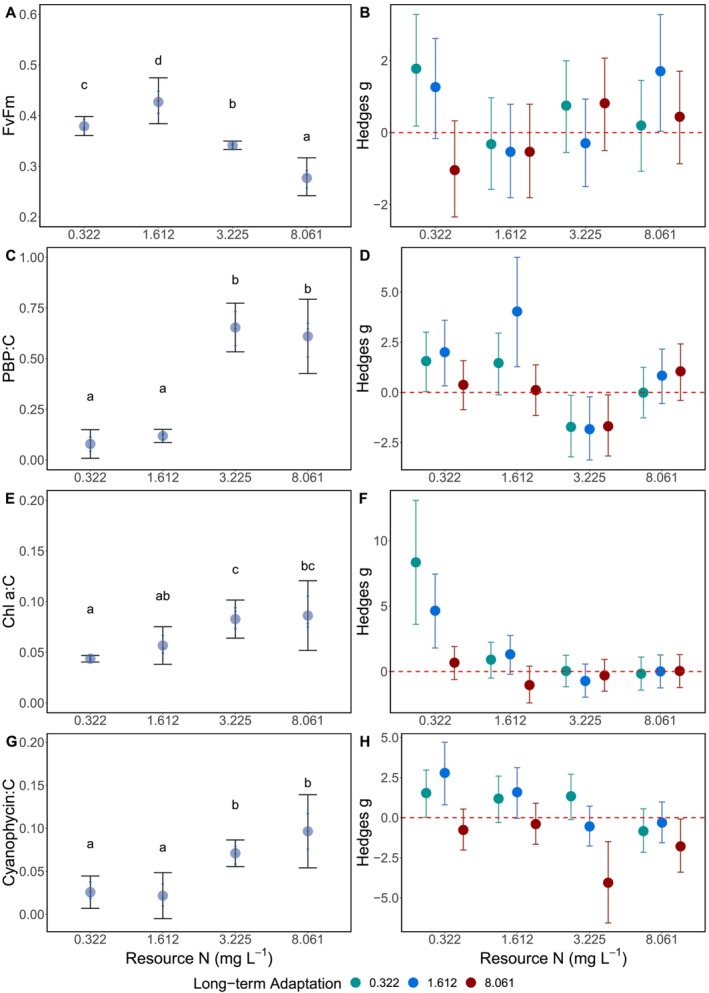
*Dolichospermum* population photosynthetic efficiency (FvFm; A), phycobilin pigment standardised to C (PBP:C; C), chlorophyll a standardised to C (Chl a:C; E), and cyanophycin standardised to C (Cyano:C; G) grown across an N supply gradient (mg L^−1^) for the short‐term adaptation experiment. Letters indicate significant grouping using the Sidak post hoc comparisons. *Dolichospermum* populations adapted to different N supplies (0.322 mg L^−1^; green, 1.612 mg L^−1^; blue, and 8.061 mg L^−1^; red) long term were compared to the short‐term adaptation using the Hedges *g* effect size and 95% confidence intervals for FvFm (B), PBP:C (D), Chl a:C (F), and Cyano:C (H). If 95% confidence interval overlaps with the 0 (dashed red line), it indicates the long‐term adaptation was not different from the short‐term adaptation grown under the corresponding N supply concentrations. If the 95% confidence interval does not overlap with the red dashed line, it indicates the long‐term adaptation response differed from the short‐term adaptation response. Negative effect sizes indicate the long‐term adaptation response was less than the short‐term adaptation response, and positive effect sizes indicate the long‐term adaptation response was greater than the short‐term adaptation response.

FvFm differed among all short‐term adapted populations to different N‐supplies (*F*_3,7_ = 49.43, *p* < 0.001; Figure 3A). Populations grown in 1.612 mg L^−1^ N had the highest FvFm, while populations grown in 8.061 mg L^−1^ N had the lowest FvFm. Long‐term adaptation to different N‐supplies did not systematically affect the FvFm (Figure 3B). Populations grown in lower N‐supplies (0.322 and 1.612 mg L^−1^ N) had three times fewer C‐standardised PBPs compared to the populations grown in high N treatments (3.225 and 8.061 mg L^−1^ N; *F*_3,7_ = 105.44, *p* < 0.001; Figure 3C). *Dolichospermum* adapted to low N supplies long term had more PBPs when grown under these conditions, and less PBP in the 3.225 mg L^−1^ N than the short‐term adapted populations (Figure 3D). Populations grown in the two lowest N‐supplies short term had approximately 40% less C‐standardised Chl a compared to populations grown in 3.225 mg L^−1^ N (*F*_3,7_ = 24.63, *p* < 0.001; Figure 3E). The populations adapted to the two lowest N‐supplies longterm had more Chl a compared to short‐term population when grown in 0.322 mg L^−1^ N (Figure 3F). Populations adapted to the two lowest N‐supplies short term had approximately 30% less C‐standardised cyanophycin compared to *Dolichospermum* grown the higher N‐supplies (*F*_3,7_ = 27.12, *p* < 0.001; Figure 3G). The population adapted to 1.612 mg L^−1^ N long term had more cyanophycin than the short‐term population grown in the 0.322 mg L^−1^ N (Figure 3H).

**FIGURE 4:**
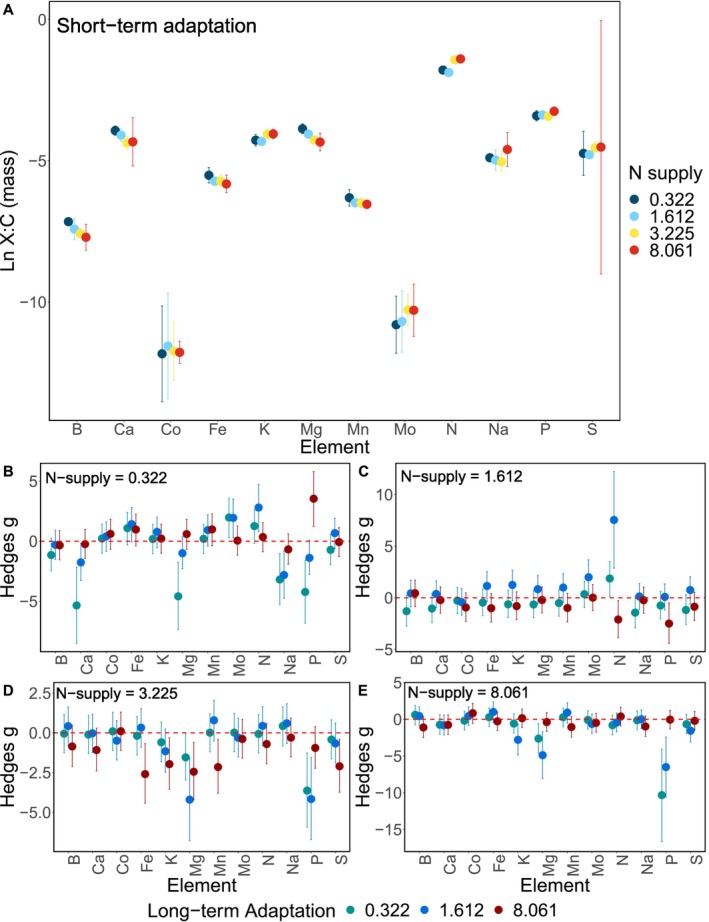
The natural log transformed ratios of the boron (B), calcium (Ca), cobalt (Co), iron (Fe), potassium (K), magnesium (Mg), manganese (Mn), molybdenum (Mo), nitrogen (N), sodium (Na), phosphorus (P), and sulphur (S) to C (*X*:C by mass; A) of *Dolichospermum flosaquae* of short‐term adapted populations to different N supplies of 0.322 mg L^−1^ (dark blue), 1.612 mg L^−1^ (light blue), 3.255 mg L^−1^ (yellow), and 8.081 mg L^−1^ (red). Significant differences between short‐term adaptation within a *X*:C ratio are reported in Table 1. *Dolichospermum* populations adapted to different N supplies (0.322 mg L^−1^; green, 1.612 mg L^−1^; blue, and 8.061 mg L^−1^; red) long term were compared to the short‐term adaptation using the Hedges *g* effect size and 95% confidence intervals for all *X*:C ratios grown in 0.322 mg L^−1^ (B), 1.612 mg L^−1^ (C), 3.255 mg L^−1^ (D), and 8.081 mg L^−1^ (E). If the 95% confidence interval does not overlap the red dashed line, it indicates the long‐term adaptation response differed from the short‐term adaptation response. Negative effect sizes indicate the long‐term adaptation response was less than the short‐term adaptation response, and positive effect sizes indicate the long‐term adaptation response was greater than the short‐term adaptation response.

Short‐term adaptation to different N‐supplies affected the ionomic composition of *Dolichospermum* populations. All data are reported as C‐standardised ALRs. We found the two lowest N‐supplies had less N than the higher N supplies (Table 1; Figure 4A). *Dolichospermum* adapted to 3.255 mg L^−1^ N for 27 days had more K and S than populations grown in 1.612 mg L^−1^ N (Table 1; Figure 4A). *Dolichospermum* adapted short term to the two lowest N‐supplies had more B, Ca, and Mg than the higher N‐supplies (Table 1; Figure 4A).

**TABLE 1:** Generalised linear model *F* and *P* values for the natural log transformed element:carbon (*X*:C) for the short‐term adapted *Dolichospermum* populations to different nitrogen supply. Untransformed element: Carbon averages with significant differences indicated by different letters. Data are graphed in Figure 4.

Element:Carbon	*F*	*P*	N supply (mg L^−1^)			
			0.322	1.612	3.255	8.081
**Boron**	28.13	0.001	7.81 × 10^−4^ b	6.06 × 10^−4^ ab	5.18 × 10^−4^ a	4.55 × 10^−4^ a
**Calcium**	8.32	0.02	0.0197 b	0.0167 b	0.0128 a	0.0137 ab
Cobalt	0.10	0.96	9.93 × 10^−6^ a	1.15 × 10^−5^ a	9.11 × 10^−6^ a	7.72 × 10^−6^ a
Iron	2.53	0.18	0.0041 a	0.0033 a	0.0033 a	0.0030 a
**Potassium**	24.08	0.006	0.0141 ab	0.0134 a	0.0174 b	0.0175 b
**Magnesium**	19.08	0.004	0.0210 b	0.0173 b	0.0141 a	0.0131 a
Manganese	2.033	0.25	1.85 × 10^−3^ a	1.53 × 10^−3^ a	1.52 × 10^−3^ a	1.45 × 10^−3^ a
Molybdenum	1.22	0.39	2.35 × 10^−5^ a	2.42 × 10^−5^ a	3.59 × 10^−5^ a	3.56 × 10^−5^ a
**Nitrogen**	1150.9	< 0.001	0.167 a	0.153 a	0.241 b	0.248 b
Sodium	2.42	0.17	7.51 × 10^−3^ a	6.95 × 10^−3^ a	6.62 × 10^−3^ a	1.03 × 10^−2^ a
Phosphorus	4.33	0.07	0.033 a	0.034 a	0.033 a	0.039 a
**Sulphur**	7.01	0.03	8.83 × 10^−3^ ab	8.34 × 10^−3^ a	1.08 × 10^−2^ b	1.10 × 10^−2^ ab

Comparing the long‐ and short‐term adapted populations, long‐term adaptation to 0.322 mg L^−1^ N caused *Dolichospermum* populations to have less Ca, Mg, Na, P, and more Mo and N when grown in 0.322 mg L^−1^ N (Figure 4B) and more N when grown in 1.612 mg L^−1^ N (Figure 4C). *Dolichospermum* adapted long term to 0.322 mg L^−1^ N and grown in either 3.225 or 8.061 mg L^−1^ N resulted in less Mg and P (Figure 4D,E). Long‐term adaptation to 1.612 mg L^−1^ N caused *Dolichospermum* populations to have less Ca, Na, and more Mo when grown in 0.322 mg L^−1^ N (Figure 4B), more Mo and N when grown in 1.612 mg L^−1^ N (Figure 4C), and less Mg and P when grown in 3.225 and 8.061 mg L^−1^ N (Figure 4D,E). The population adapted to 1.612 mg L^−1^ long term and grown in 8.061 mg L^−1^ N had less K (Figure 4E). Long‐term adaptation to 8.061 mg L^−1^ N only affected some of the elemental composition when grown in 0.322 and 3.225 mg L^−1^ N (Figure 4B–E). *Dolichospermum* population adapted to 8.061 mg L^−1^ N long term had more P when grown in 0.322 mg L^−1^ N (Figure 4B) and less K, Fe, Mg, Mn, and S when grown in 3.225 mg L^−1^ N (Figure 4D).

**FIGURE 5:**
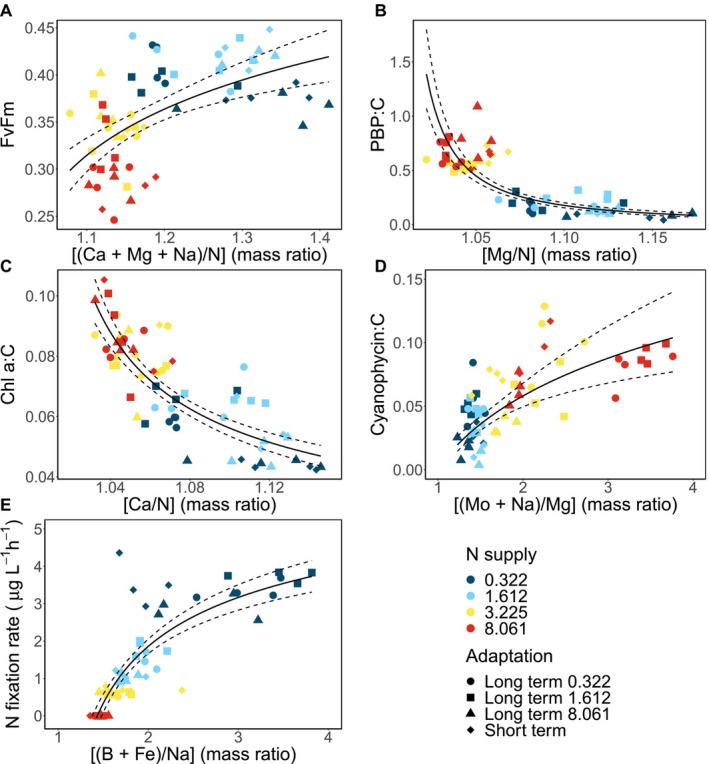
Log regression of the significant elements identified from the partial least squares regression to the log photosynthetic efficiency (FvFm; A) log phycobilin pigments standardised to carbon (PBP:C; B) log chlorophyll a standardised to C (Chl a:C; C), log cyanophycin standardised to C (cyanophycin:C: D), and cumulative nitrogen fixation rates (μg L^−1^ h^−1^; E). Populations were grown in resource N supply of 0.322 mg L^−1^ (dark blue), 1.612 mg L^−1^ (light blue), 3.255 mg L^−1^ (yellow), and 8.081 mg L^−1^ (red). Diamond shapes are the short‐term adaptation experiment, and the circles, squares, and triangles are the long‐term adaptation to resource N concentrations of 0.322, 1.612, and 8.061 mg L^−1^, respectively.

The [(Ca + Mg + Na)/N] ratio best explained the variation in the FvFm (Table S3). There was a positive log–log correlation between the [(Ca + Mg + Na)/N] ratio and FvFm (*F*_1,60_ = 28.51 *p* < 0.001, *r* ^2^ = 0.31; Figure 5A). Regardless of adaptation, the populations grown in 3.225 and 8.061 mg L^−1^ N had a [(Ca + Mg + Na)/N] ratio below 1.2, while the majority of the 0.322 and 1.612 mg L^−1^ N had a [(Ca + Mg + Na)/N] ratio above 1.2 (Figure 5A). We identified Mg and N as the most important elements to explain the variation in the phycobilin pigments (Table S3). We found a negative log–log correlation between the Mg:N and C‐standardised phycobilin pigments (*F*_1,60_ = 167.6, *p* < 0.001, *r* ^2^ = 0.73; Figure 5B). *Dolichospermum* populations grown in the 3.225 and 8.061 mg L^−1^ N had more phycobilin pigments and lower Mg:N ratios compared to the 0.322 and 1.612 mg L^−1^ N populations (Figure 5B). The best predictor to explain the variation in Chl a was the Ca:N ratio (Table S3). We found a negative log–log relationship between the Ca:N and Chl a, with *Dolichospermum* populations grown in the 3.225 and 8.061 mg L^−1^ N having Ca:N below 1.06 (*F*_1,60_ = 125.7, *p* < 0.001, *r* ^2^ = 0.67; Figure 5C). The [(Mo + Na)/Mg] ratio best explained the variation in the N‐storage molecule, cyanophycin (Table S3). We found a positive log–log relationship between the [(Mo + Na)/Mg] ratio and cyanophycin with *Dolichospermum* populations grown in 0.322 and 1.612 mg L^−1^ N having lower [(Mo + Na)/Mg] and less cyanophycin (*F*_1,60_ = 38.95 *p* < 0.001, *r* ^2^ = 0.38; Figure 5D). The [(B + Fe)/Na] ratio best explained the cumulative N‐fixation rates (Table S3). We identified a positive relationship between [(B + Fe)/Na] and N‐fixation rates with *Dolichospermum* populations grown in 0.322 and 1.612 mg L^−1^ N having higher [(B + Fe)/Na] ratios than the other populations (*F*_1,60_ = 49.5, *p* < 0.001, *r* ^2^ = 0.71; Figure 5E).

## 4 Discussion

Both short‐ and long‐term adaptations to different N‐supplies caused numerous physiological and ionomic shifts. Our hypothesis that short‐term adaptation to different N‐supplies would result in unique ionome profiles was supported (Figure 4A; Table 1; and Table S4). Although only one of our predicted changes, higher B:C, occurred in the N‐deficient populations. We identified some unpredicted shifts (e.g., higher Ca:C, and Mg:C in N‐deficient conditions) linked to changes in eco‐physiological traits. We also found support for the hypothesis that long‐term adaptation to low N‐supplies will enhance the expression of the N‐fixation phenotype caused by the continuous N‐fixation demand. For example, populations adapted to 0.322 and 1.612 mg L^−1^ N long term had more Chl a, cyanophycin, and higher B:C and Mo:C, which could prime the populations for N‐fixation. Overall, some of the ionomic eco‐physiological traits correlations were predicted from enzyme requirements, such as increased Mo:C in N‐fixing populations; however, many of the ionomic physiological trait correlations to differences in N‐supply could not be predicted based solely on changes in enzyme requirements.

### 4.1 Physiological and Ionome Changes in Photosynthesis

We found correlations between light‐harvesting molecules and elemental composition. Chl a and PBP negatively correlated with Ca:N and Mg:N, respectively. However, FvFm positively correlated with the [(Ca + Mg + Na)/N] ratio. The negative relationships between the light‐harvesting pigments and Ca:N and Mg:N could be caused by the increased Ca:C and Mg:C in the N‐fixing populations (Figure 4A). FvFm measures the proportion of open PSII reaction centres that can accept electrons for light harvesting to closed reaction centres, in which electrons are dissipated as heat (Parkhill, Maillet, and Cullen 2001). Low FvFm occurs in populations grown under nutrient‐stress or high light intensity, which causes more electrons to dissipate as heat than under non‐stressed conditions (Parkhill, Maillet, and Cullen 2001). However, PBP cause a high dark‐adapted fluorescence that decreases the variable fluorescence (Fv), resulting in an artificially low photosynthetic yield (Campbell et al. 1998). Thus, the differences in the relationships between FvFm and light‐harvesting pigments may be an artefact in the FvFm measurement, because low resource N‐supplies had fewer PBPs and the appearance of a higher quantum yield.

*Dolichospermum* populations adapted (short term; 27d) to the two highest N‐supplies had higher N:C and K:C than the populations grown in low N‐supplies. The proteins that make up the phycobilisome are the largest pool of soluble proteins within cyanobacteria (Tandeau De Marsac et al. 2001) and can act as an N‐reserve in high N‐conditions that are degraded during periods of N‐stress (Wang et al. 2021), which likely explains the differences in N:C between the high and low N‐supply populations. Photosystem I (PSI) and PSII depend on a voltage gradient to move electrons across the thylakoid membrane. In cyanobacteria, K ions promote that voltage gradient (Checchetto et al. 2013); consequently, more light‐harvesting pigments and thylakoid membranes could cause a higher K quota. Furthermore, long‐term adaptation to 8.061 mg L^−1^ N had lower K:C and fewer phycobilin pigments than the short‐term adaptation population grown in 3.225 mg L^−1^ N, highlighting the connections between K and photosynthetic capacity.

### 4.2 Physiological and Ionome Changes in N‐Fixation

Consistent with prior observations (e.g., Raven 2012; Osburn, Wagner, and Scott 2021), a growth cost of N‐fixation was apparent, as populations with the highest cumulative N‐fixation rate did not produce as much biomass as populations grown high N‐supplies. This growth trade‐off may be associated with demographic shifts in heterocystous cyanobacteria (Grover et al. 2020) and/or increased ribosome demands to maintain active nitrogenases (Raven 2012). Short‐term adapted populations grown in the two lowest N‐supplies had fewer light‐harvesting pigments and less cyanophycin, which is physiologically similar to non‐diazotrophic cyanobacteria displaying classic N‐limitation symptoms, even though the N:C composition is not as plastic as the non‐N‐fixing taxa (Wagner et al. 2021; Wang et al. 2021). Additional ionomic changes in N‐fixing *Dolichospermum* populations include higher B:C and Ca:C than non‐N‐fixing populations. Boron is an essential element in heterocystous cyanobacteria that stabilises the glycolipids in the outer envelope of the heterocyst (Bonilla, Garcia‐Gonzalez, and Mateo 1990). Populations of *Dolichospermum* grown in B‐limited conditions have lower nitrogenase activity caused by the destabilisation of the glycolipid membrane that increases O_2_ diffusion (Gracia‐Gonzalez, Mateo, and Bonilla 1988). *Dolichospermum* populations grown in Ca‐limited environments have reduced N‐fixation rates (Bonilla, Bolanos, and Mateo 1995), which may be caused by Ca involvement in heterocyst development, increased Ca demands in the heterocysts compared to vegetative cells (Shi et al. 2006), and the Ca‐dependent uptake of B (Bonilla, Bolanos, and Mateo 1995; El‐Zahraa and Zaki 1999).

Our results confirm that cyanobacteria adapted to low N supply promote diazotrophy and can increase their N‐fixation rates and N:C ratios under short acclimation periods (Osburn, Wagner, and Scott 2021). We found that this pattern is maintained even after 45 months of adaptation in the 1.612 mg L^−1^ N population (Figures 2D and 4B,C). Additionally, *Dolichospermum* adapted to the two lowest N‐supplies long term had higher Mo:C than the short‐term adapted populations (Figure 4B,C). Nitrogenase can range between 3% and 10% of the protein pool (Glass et al. 2010; Raven 2012), and the demands for Mo are approximately 125 times higher for diazotrophy than nitrate uptake (Raven 1988). Since Mo can limit phytoplankton growth in lakes (Glass et al. 2012), populations adapted to low N supplies that rely on N‐fixation may store Mo to ensure continued growth through diazotrophy.

We found more Chl a in *Dolichospermum* populations that were long‐term adapted to the two lowest N‐supplies. Chl a is an essential component of PSI and PSII, with PSI containing most of the Chl a (Rakhimberdieva et al. 2001). In the marine diazotroph, *Trichodesmium*, the ratio of PSI:PSII is plastic depending on the environmental conditions but varies between 2.5 and 4 (Levitan et al. 2010). Most of the ATP that fuels N‐fixation is produced within the heterocysts by PSI during light reactions (Milligan et al. 2007). Additionally, PSI can photo‐catalyse the reduction of oxygen to water, which is a mechanism to maintain anoxic conditions within the heterocyst (Milligan et al. 2007). Therefore, *Dolichospermum* populations adapted to 0.322 and 1.612 mg L^−1^ N long term possibly have more plasticity in the PSI:PSII ratio to increase the PSI in the heterocysts, providing more energy and oxygen protection for N‐fixation.

Interestingly, Mg:C ratios were higher in the short‐term adapted populations grown in the two lowest N‐supplies than the populations grown in high N‐supplies. Essential roles of Mg include a co‐factor in Chl a and binding to phosphates in ATP and nucleic acids (Pohland and Schneider 2019). We expect N‐fixing populations to have higher P‐demands because of the increased ATP requirement for N‐fixation and the ribosome synthesis to maintain active nitrogenase enzymes (Raven 2012). However, we found populations adapted to the two lowest N‐supplies short term had less Chl a and similar P:C ratios than populations grown in higher N‐supplies. A possible explanation is that Mg ions can act similarly to the K ions in creating the electrochemical differences across the thylakoid membrane (Shcolnick and Keren 2006). Thus, *Dolichospermum* populations adapted to low N‐supplies short term used more Mg ions to create the electrochemical gradient across the thylakoid membrane, while the populations grown in high N‐supplies used more K ions in creating the electrochemical gradient. *Dolichospermum* adapted long term to the two lowest N‐supplies had similar K:C and decreased Mg:C and P:C than short‐term adapted populations. Therefore, we predict the decrease in Mg:C was caused by lower P:C ratios, which decreases Mg demand for P‐binding and are not needed to create an electrochemical gradient across the thylakoid membrane.

## 5 Conclusions

We observed considerable plasticity in the elemental physiology of the freshwater cyanobacteria, *Dolichospermum* to variable N‐supplies at both short (27 days) and long (45 months) timescales. Indeed, numerous elements responded to variable N‐supply based on their known roles in the ecophysiology of heterocystous diazotrophs. Short‐term adaption to low N‐supply resulted in ionomic changes that appear to be driven by nitrogenase demand and heterocyst formation. While this pattern of ionomic change was observed with long‐term adaptation to low N‐supply (i.e., high Mo:C), other physiological elemental connections were not predicted. For example, long‐term adapted diazotrophic N‐fixing phenotype decreased many of the ion balance elements (Ca, Mg, and Na) that are involved in photosynthesis when grown in diazotrophic conditions than the short‐term adapted population. However, the long‐term adapted diazotrophic phenotype ionomic profile was not constant across N‐supplies. Thus, the ionomic composition of *Dolichospermum* is dependent on the current and historical nutrient conditions. Overall, our results highlight that using first principles to predict elemental composition from changes in physiological pools can be helpful in understanding changes in organismal demand and storage. However, solely basing elemental composition predictions off of known enzyme changes (i.e., nitrogenase) may under appreciate the whole ionomic rearrangement caused by differences in physiology. Therefore, we suggest continuing to explore the physiological ionomic interactions to see if generalisable patterns can be observed in phylogenetically distinct organisms.

## Author Contributions

N.D.W., C.P., P.D.J., and J.T.S. designed the study, N.D.W., F.S.O., J.W., and C.P. collected the data, N.D.W., C.J.R., and C.P. analysed the data. N.D.W. wrote the first draft with revisions from all co‐authors.

### Peer Review

The peer review history for this article is available at https://www.webofscience.com/api/gateway/wos/peer‐review/10.1111/ele.70051.

## Supporting information


Data S1.

## Data Availability

The data and corresponding code supporting the findings of this study are publicly available on FigShare. https://doi.org/10.6084/m9.figshare.24312436.v5.
