# Supplementary material for: Current and Historical Resource Nitrogen Supply Affects the Eco‐Physiological Traits and the Ionome of a Diazotrophic Cyanobacterium
Source: Ecol Lett. 2024 Dec 31;28(1):e70051. doi: 10.1111/ele.70051 (PMC11687340; doi:10.1111/ele.70051)
Supplement: Supplementary file 1 — Data S1. [file ELE-28-0-s001.docx]

**Current and historical resource nitrogen supply affects the eco-physiological traits and the ionome of a diazotrophic cyanobacterium**

**Supplementary Information**

Nicole D. Wagner^1^, Clay Prater^2,3^, Caleb J. Robbins^4,5^, Felicia S. Osburn^6^, Jingyu Wang^4,7^, Punidan D. Jeyasingh^2^, J. Thad Scott^4,5^

^1^ Department of Biological Sciences, Oakland University, Rochester, Michigan, USA

^2^ Department of Integrative Biology, University of Oklahoma, Stillwater, Oklahoma, USA

^3^ Department of Biological Science, University of Arkansas, Fayetteville, Arkansas, USA

^4^ Center for Reservoir and Aquatic System Research, Baylor University, Texas, USA

^5^ Department of Biology, Baylor University, Texas, USA

^6^ Department of Biology, University of Central Arkansas, Arkansas, USA

^7^ College of Environmental Science and Environmental Engineering, Zhejiang University of Water Resources and Electric Power, Hangzhou, China

**Author Statement:** NDW, CP, PDJ, and JTS designed the study, NDW, FSO, JW and CP collected the data, NDW, CJR, and CP analyzed the data. NDW wrote the first draft with revisions from all coauthors.

**Data availability statement**: Data are available on FigShare. DOI: https://doi.org/10.6084/m9.figshare.24312436.v4

**Supplementary Methods**

*Photosynthetic efficiency*

We calculated photosynthetic efficiency, the FvFm ratio, by removing 4 ml of a 10 h dark acclimated culture and measuring the *in vivo* chlorophyll a (Chla) fluorescence. We then added 50 µL of 3 mM Diuron (DCMU; a PSII inhibitor), incubated the sample for 60 s, and remeasured the *in vivo* Chla fluorescence. The FvFm ratio was calculated as previously described by (Parkhill et al., 2001).

*Two source-mixing model for N-fixation rate*

The δ ^15^N of the final sample was defined in a two source-mixing model

| $\delta{}_{1}^{15}N=\left( \delta{}_{1}^{15}{N_{added}}\times f_{added} \right)+(\delta{}_{1}^{15}{N_{fix}}\times f_{fix}$) | (1) |
| --- | --- |

Where δ ^15^N_added_, and δ ^15^N_fix_ are the isotopic signatures of the nitrate-N added (100 ‰), and the δ ^15^N of the atmosphere (0 ‰). The *f* variables are the fractional contribution of the N added and fixed N to the final N concentrations. Thus, the sum of f_added_, and f_fix_ are assumed to be 1. Since we know the initial *D. flos-aquae* isotopic signature was (0 ‰) this simplifies the equation to

| ${-f}_{\delta{}_{1}^{15}N}=\frac{\left( \delta{}_{1}^{15}{N_{final}}- \delta{}_{1}^{15}{N_{initial}} \right)}{\left( \delta{}_{1}^{15}{N_{fix}}- \delta{}_{1}^{15}{N_{added}} \right)}$ | (2) |
| --- | --- |
| $N_{fix}=1- f_{\delta{}_{1}^{15}N}$ | (3) |

Where both the isotopic signature of the ^15^N_initial_ and ^15^N_fix_ are 0 ‰, ^15^N­_added_ is 100 ‰. Our 2-source mixing model assumes no isotopic fractionation. Nitrate uptake-induced fractionation can vary 4 to 19 ‰ in cyanobacteria (Bauersachs et al., 2009), thus our results may have higher cumulative N-fixation rates in our populations grown in high N:P condition.

*Cyanophycin analysis*

Briefly, cells collected on cellulose acetate filters (25mm 0.45µm pore size; Whatmans) were washed with 2 mL of 0.1 M Tris-HCl buffer at pH 7.0. The filter was removed, and the slurry under went three freeze-thaw cycles to lyse cells. After, the slurry was then centrifuged at 7000 RPM for 10 minutes and the supernatant was discarded. Cyanophycin was extracted by two treatments using 0.5 mL of 0.1 M HCl for 30 minutes at room temperature. The resulting supernatant was pooled and neutralized with 0.1 M NaOH and centrifuged at 7000 RPM for 10 minutes. The purified cyanophycin pellet was resolubilized in 1.5 mL of 0.1 M HCl. The soluble cyanophycin was measured using the Bradford dye assay and concentrations calculated using albumin as the standard.

*Statistical analysis*

*Generalized least squares regression*

We initially evaluated ordinary least squares regression models and found residual heteroskedasticity across resource N-supply for all the physiological and ionome responses except cumulative N-fixation, so we used the varIdent function in nlme::gls() to weight variance by N-supply (Pinheiro et al., 2022; Zuur et al., 2009) in all analyses except cumulative N-fixation. We log-transformed the biomass and FvFm to better approximate the assumptions of the generalized linear regression. The estimated response means and 95% confidence intervals were obtained for each experimental treatment using the emmeans package (Lenth, 2022). Model fit was assessed using the performance package (Lüdecke et al., 2021). Sidak post hoc comparisons assessed significant differences among resource N-supply within the short-term adaptation treatments.

*Partial least squares regression*

We used a partial least squares regression (PLSR), which is appropriate for co-linear data (Carrascal et al., 2009) to examine the correlations between physiological traits and elemental composition. To select the elements that correlate with the physiological responses, we used the ipw_pls function in the plsVarSel package (Mehmood et al., 2012). This function uses an iterative elimination procedure to determine the most important variables within the PLSR. The number of iterations was set to 500 and the threshold correlation coefficient was set to 0.1 using the regression coefficient filter. The selected elements were then included in the plsr function within the pls package (Liland et al., 2022). We used leave-one-out cross-validation to identify the number of PLS components that minimized the lowest root mean square error in the prediction (RMSEP). The total variation explained between the selected elemental and the physiological variable was recorded for the number of components, and the resulting R^2^ of the model was determined using the R2() function within the pls package.

**References**

Bauersachs, T., Schouten, S., Compaore, J., Wollenzien, U., Stal, L. J., & Sinninghe Damste, J. S. (2009). Nitrogen isotopic fractionation associated with growth on dinitrogen gas and nitrate by cyanobacteria. *Limnology and Oceanography*, *54*(4), 1403–1411. https://doi.org/10.4319/lo.2009.54.4.1403

Carrascal, L. M., Galván, I., & Gordo, O. (2009). Partial least squares regression as an alternative to current regression methods used in ecology. *Oikos*, *118*(5), 681–690. https://doi.org/10.1111/j.1600-0706.2008.16881.x

Lenth, R. (2022). *emmeans: Estimated Marginal Means, aka Least-Squares Means. R package version 1.8.3*. <https://CRAN.R-Project.Org/Package=emmeans>.

Liland, K., Mevik, B., & Wehrens, R. (2022). *pls: Partial Least Squares and Principal Component Regression. R package version 2.8-1*. <https://CRAN.R-Project.Org/Package=pls>.

Lüdecke, D., Ben-Shachar, M., Patil, I., Waggoner, P., & Makowski, D. (2021). performance: An R Package for assessment, comparison and testing of statistical models. *Journal of Open Source Software*, *6*(60), 3139. https://doi.org/10.21105/joss.03139

Mehmood, T., Liland, K. H., Snipen, L., & Sæbø, S. (2012). A review of variable selection methods in Partial Least Squares Regression. *Chemometrics and Intelligent Laboratory Systems*, *118*, 62–69. https://doi.org/10.1016/j.chemolab.2012.07.010

Parkhill, J.-P., Maillet, G., & Cullen, J. J. (2001). Fluorescence-based maximal quantum yield for PSII as a diagnostic of nutrient stres. *J. Phycol*, *37*, 517–529.

Pinheiro, J., Bates, D., & R Core Team. (2022). *nlme: Linear and Nonlinear Mixed Effects Models. R package version 3.1-161*. <https://CRAN.R-Project.Org/Package=nlme>. <https://CRAN.R-project.org/package=nlme>

Zuur, A., Ieno, E., Walker, N., Saveliev, A., & Smith, G. (2009). *Mixed effect models and extensions in ecology with R*.


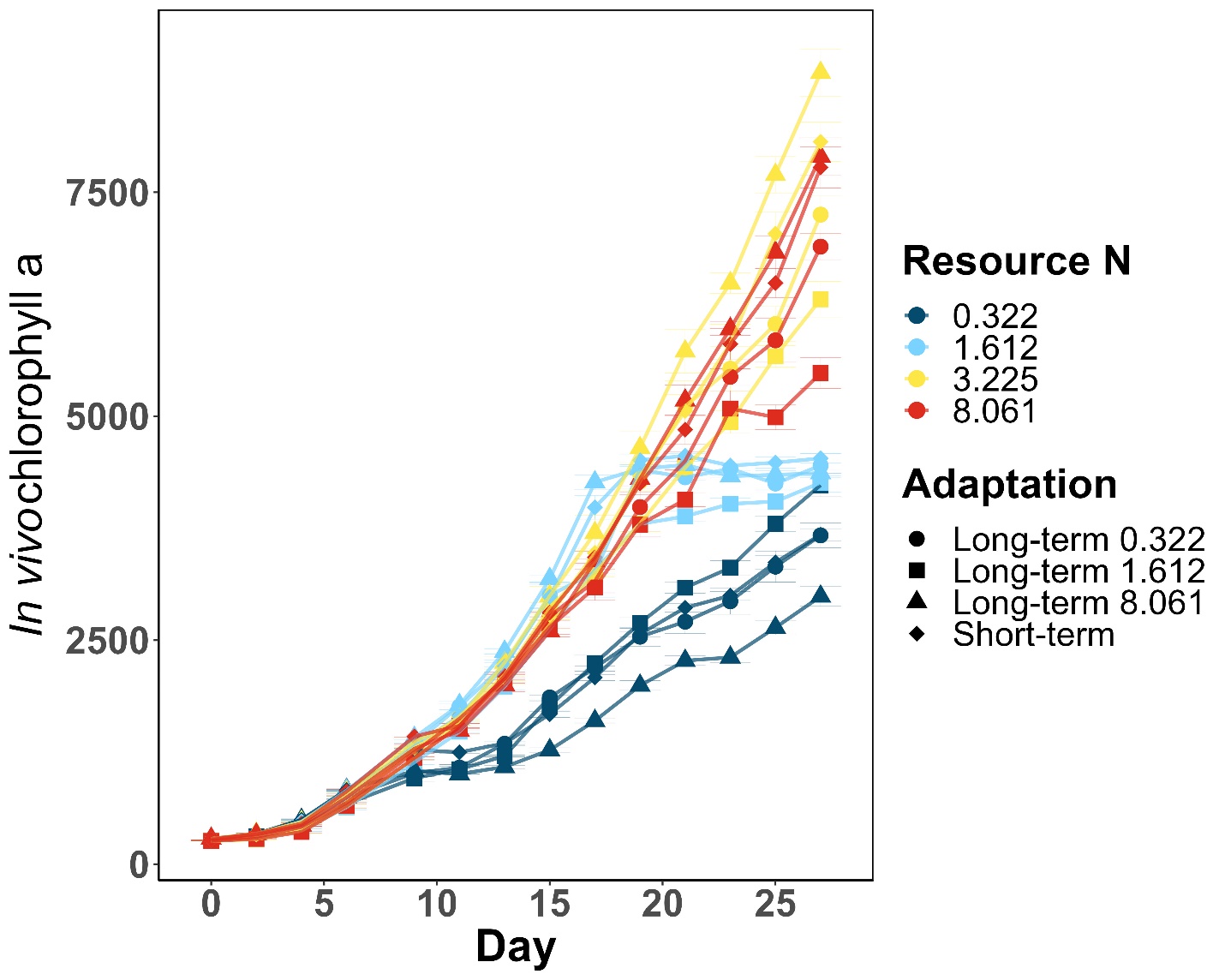


**Figure S1:** *In vivo* chlorophyll a fluorescence of batch cultures throughout the 27-day common garden experiment. Short- and long-term adapted populations were grown in 0.322 (dark blue), 1.612 (light blue), 3.255 (yellow), 8.061 (red) mg L^-1^ N. Long-term adapted populations to 0.322, 1.612, and 8.061 mg L^-1^ N are represented by circles, squares, triangles, respectively. Diamond shape data points are the short-term adaptation experiment.

**Table S1:** Concentration of major elements in our stock 0.5x BG 11 media and in the short- and long-term adapted experimental 0.05x N-Free BG-11 media. * Same concentration for all N-supplies.

| **Element** | **0.5x BG-11 (mg L^-1^)** | **0.05x BG-11 N Treatment** | **0.05x BG-11 (mg L^-^)** |
| --- | --- | --- | --- |
| **N** | 123.368 | 0.322 | 0.322 |
|  |  | 1.162 | 1.162 |
|  |  | 3.025 | 3.025 |
|  |  | 8.061 | 8.061 |
| **P** | 3.565 | * | 0.357 |
| **Mg** | 3.640 | * | 0.365 |
| **Ca** | 4.810 | * | 0.481 |
| **Fe** | 0.586 | * | 0.059 |
| **Na** | 206.727 | 0.322 | 0.971 |
|  |  | 1.162 | 3.089 |
|  |  | 3.025 | 5.409 |
|  |  | 8.061 | 13.679 |
| **K** | 8.990 | * | 0.899 |
| **S** | 4.827 | * | 0.483 |
| **B** | 0.249 | * | 0.025 |
| **Mn** | 0.247 | * | 0.025 |
| **Zn** | 0.025 | * | 0.003 |
| **Mo** | 0.077 | * | 0.008 |
| **Cu** | 0.010 | * | 0.001 |
| **Co** | 0.005 | * | 5 x 10^-4^ |

**Table S2:** Detection limits for the elements analyzed on the inductively coupled plasma optical emissions spectroscopy (ICP-OES).

| Element | Limit of Detection (µg L^-1^) |
| --- | --- |
| B | 1.14 |
| Ca | 5.19 |
| Co | 0.174 |
| Fe | 1.51 |
| K | 1.50 |
| Mg | 1.54 |
| Mo | 0.522 |
| Mn | 0.081 |
| Na | 13.16 |
| P | 3 |
| S | 8.27 |

**Table S3:** Partial least squares regression between physiological responses and ionome for populations grown in all resource N supply treatments regardless of long-term adaptation. Regression coefficient and weights for elements associated with physiological responses.

| **Physiological Response** | **Number of Components** | **R^2^** | **RMSEP** | **Significant Elements** | **Element weight within model** | **Total Variance explained by components** |
| --- | --- | --- | --- | --- | --- | --- |
| FvFm | 4 | 0.70 | 0.028 | Ca | 0.20 | 74.81 |
|  |  |  |  | Mg | 0.29 |  |
|  |  |  |  | N | 0.25 |  |
|  |  |  |  | Na | 0.25 |  |
| Phycobilin pigments | 2 | 0.80 | 0.114 | Mg | 0.32 | 82.10 |
|  |  |  |  | N | 0.68 |  |
| Chlorophyll a | 2 | 0.71 | 0.009 | Ca | 0.43 | 73.05 |
|  |  |  |  | N | 0.57 |  |
| Cyanophycin | 1 | 0.54 | 0.019 | Mg | 0.30 | 58.53 |
|  |  |  |  | Mo | 0.38 |  |
|  |  |  |  | Na | 0.32 |  |
| N-fixation rates | 2 | 0.75 | 0.64 | B | 0.39 | 78.38 |
|  |  |  |  | Fe | 0.38 |  |
|  |  |  |  | Na | 0.23 |  |

**Table S4:** The average molar C:N:P:Mg:K:Ca:Na:S:Fe:B:Mn:Mo:Co ratios of *Dolichospermum* populations for the short-term and long-term adaptation grown in different nitrogen (mg L^-1^) supplies.

| Short-term adaptation N supply | | C | N | P | Mg | K | Ca | Na | S | Fe | B | Mn | Mo | Co |
| --- | --- | --- | --- | --- | --- | --- | --- | --- | --- | --- | --- | --- | --- | --- |
| 0.322 | | 78 | 11 | 1 | 0.80 | 0.33 | 0.46 | 0.30 | 0.25 | 0.07 | 0.07 | 0.03 | 2.2 x 10^-4^ | 1.6 x 10^-4^ |
| 1.612 | | 76 | 10 | 1 | 0.65 | 0.31 | 0.38 | 0.27 | 0.24 | 0.05 | 0.05 | 0.03 | 2.3 x 10^-4^ | 1.7 x 10^-4^ |
| 3.225 | | 79 | 16 | 1 | 0.55 | 0.43 | 0.30 | 0.27 | 0.32 | 0.06 | 0.05 | 0.03 | 3.6 x 10^-4^ | 1.5 x 10^-4^ |
| 8.016 | | 66 | 14 | 1 | 0.43 | 0.36 | 0.27 | 0.35 | 0.27 | 0.04 | 0.03 | 0.02 | 3.0 x 10^-4^ | 1.0 x 10^-4^ |
| Long-term adaptation | N supply | C | N | P | Mg | K | Ca | Na | S | Fe | B | Mn | Mo | Co |
| 0.322 | 0.322 | 117 | 18 | 1 | 0.80 | 0.52 | 0.43 | 0.30 | 0.35 | 0.12 | 0.08 | 0.05 | 8.9 x 10^-4^ | 2.5 x 10^-4^ |
| 0.322 | 1.612 | 87 | 12 | 1 | 0.66 | 0.32 | 0.36 | 0.24 | 0.21 | 0.06 | 0.05 | 0.03 | 3.3 x 10^-4^ | 1.4 x 10^-4^ |
| 0.322 | 3.225 | 121 | 25 | 1 | 0.56 | 0.57 | 0.47 | 0.48 | 0.45 | 0.08 | 0.07 | 0.04 | 5.6 x 10^-4^ | 2.2 x 10^-4^ |
| 0.322 | 8.016 | 123 | 25 | 1 | 0.51 | 0.61 | 0.39 | 0.63 | 0.44 | 0.09 | 0.07 | 0.04 | 5.3 x 10^-4^ | 2.1 x 10^-4^ |
| 1.612 | 0.322 | 100 | 17 | 1 | 0.85 | 0.54 | 0.43 | 0.28 | 0.40 | 0.12 | 0.09 | 0.05 | 7.9 x 10^-4^ | 2.4 x 10^-4^ |
| 1.612 | 1.612 | 75 | 12 | 1 | 0.72 | 0.38 | 0.40 | 0.28 | 0.28 | 0.07 | 0.06 | 0.03 | 4.5 x 10^-4^ | 1.2 x 10^-4^ |
| 1.612 | 3.225 | 115 | 24 | 1 | 0.55 | 0.54 | 0.44 | 0.46 | 0.42 | 0.08 | 0.07 | 0.04 | 4.5 x 10^-4^ | 1.6 x 10^-4^ |
| 1.612 | 8.016 | 104 | 22 | 1 | 0.41 | 0.44 | 0.33 | 0.55 | 0.36 | 0.07 | 0.06 | 0.04 | 3.5 x 10^-4^ | 2.0 x 10^-4^ |
| 8.016 | 0.322 | 54 | 8 | 1 | 0.63 | 0.24 | 0.31 | 0.18 | 0.18 | 0.06 | 0.04 | 0.03 | 1.7 x 10^-4^ | 1.6 x 10^-4^ |
| 8.016 | 1.612 | 85 | 11 | 1 | 0.71 | 0.33 | 0.41 | 0.29 | 0.24 | 0.06 | 0.06 | 0.03 | 2.5 x 10^-4^ | 8.9 x 10^-4^ |
| 8.016 | 3.225 | 89 | 18 | 1 | 0.48 | 0.39 | 0.29 | 0.29 | 0.28 | 0.04 | 0.05 | 0.02 | 3.4 x 10^-4^ | 1.6 x 10^-4^ |
| 8.016 | 8.016 | 66 | 14 | 1 | 0.41 | 0.36 | 0.21 | 0.28 | 0.26 | 0.04 | 0.03 | 0.02 | 2.4 x 10^-4^ | 1.5 x 10^-4^ |
